# Supplementary material for: Untargeted Lipidomics Reveal Quality Changes in High-Moisture Japonica Brown Rice at Different Storage Temperatures
Source: Foods. 2023 Nov 22;12(23):4218. doi: 10.3390/foods12234218 (PMC10706144; doi:10.3390/foods12234218)
Supplement: Supplementary file 1 [file foods-12-04218-s001.zip › foods-2720321-supplementary.pdf]

Supporting Information

For

Untargeted Lipidomics Reveals Quality Changes of High-moisture Japonica Brown Rice during Different Storage Temperatures

Lingyu Qu, Yan Zhao*, Xiangdong Xu, Yanfei Li, and Haoxin Lv

School of Food and Strategic Reserves, Henan University of Technology, Zhengzhou, 450000, PR China

Corresponding Author

* Prof. Yan Zhao (Y. Zhao).

Email: zhaoyanss10@126.com

Supporting information Figure. S1

Figure. S1

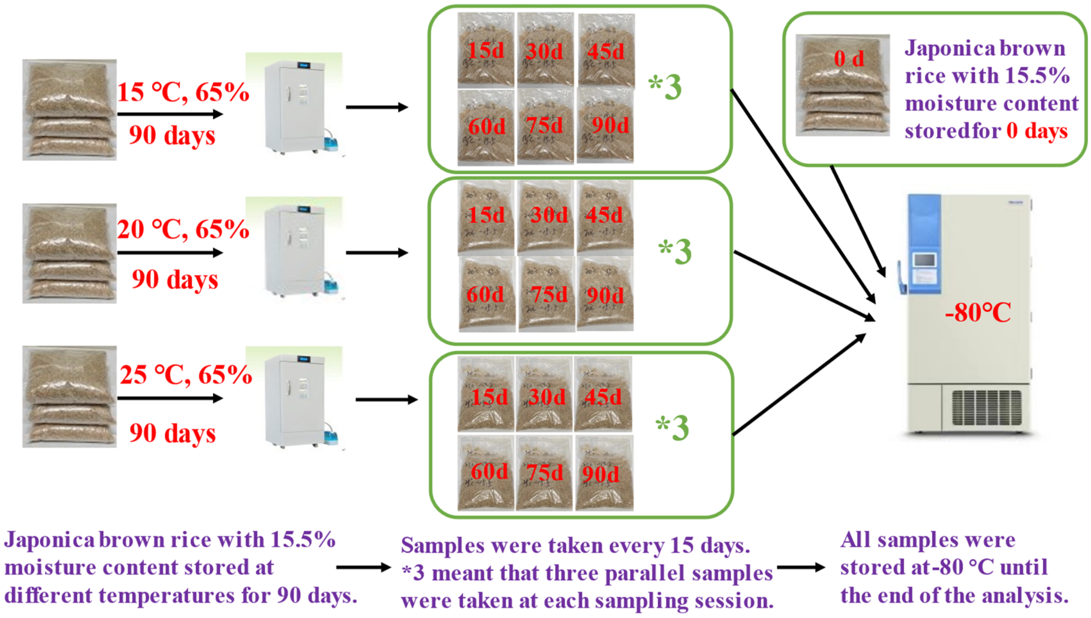

Figure. S1. The real condition of storage experimentation.
